# *catena*-Poly[[aqua­bis­(*N* ^6^-benzyl­adenine-κ*N* ^3^)copper(II)]-μ-benzene-1,4-dicarboxyl­ato-κ^2^ *O* ^1^:*O* ^4^]

**DOI:** 10.1107/S1600536811032168

**Published:** 2011-08-17

**Authors:** Wen-Bo Li

**Affiliations:** a Department of Chemistry, Dezhou University, Dezhou, Shandong 253023, People’s Republic of China

## Abstract

In the title compound, [Cu(C_8_H_4_O_4_)(C_12_H_11_N_5_)_2_(H_2_O)]_*n*_, the Cu^II^ ion is five-coordinated by two carboxyl­ate O atoms from two symmetry-related benzene-1,4-dicarboxyl­ate ligands, two N atoms from two symmetry-related *N* ^6^-benzyl­adenine ligands and one water O atom in a square-pyramidal environment. The Cu^II^ and water O atoms lie on a twofold rotation axis, and the benzene-1,4-dicarboxyl­ate ligand lies on an inversion center. The water O atom occupies the apical position and the basal plane is occupied by two O atoms and two N atoms. Each benzene-1,4-dicarboxyl­ate anion acts as a bis-monodentate ligand that binds two Cu^II^ cations, forming an infinite chain extending parallel to [001]. The *N* ^6^-benzyl­adenine ligands are attached on both sides of the chain. Neighboring chains are further inter­connected into the resulting three-dimensional supra­molecular architecture *via* O—H⋯O, N—H⋯O and N—H⋯N hydrogen bonds.

## Related literature

For examples of the use of biomolecules in metal-organic frameworks, see: An *et al.* (2009 ▶); Lee *et al.* (2008 ▶); Xie *et al.* (2007 ▶).
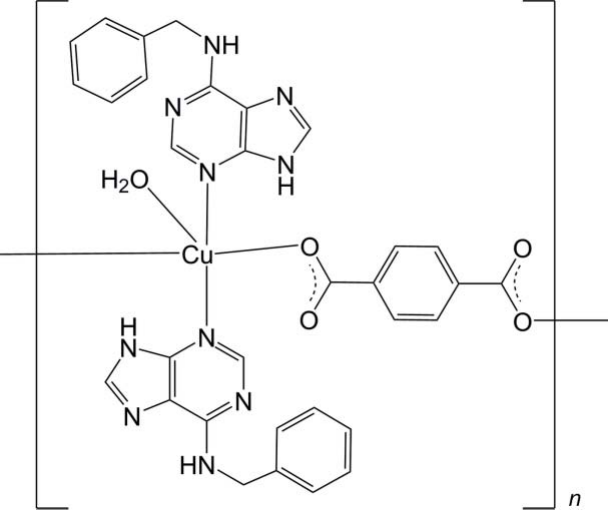


## Experimental

### 

#### Crystal data

[Cu(C_8_H_4_O_4_)(C_12_H_11_N_5_)_2_(H_2_O)]
*M* *_r_* = 696.18
Monoclinic, 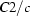

*a* = 28.171 (2) Å
*b* = 5.554 (1) Å
*c* = 22.102 (1) Å
β = 115.868 (1)°
*V* = 3111.6 (6) Å^3^
*Z* = 4
Mo *K*α radiation
μ = 0.76 mm^−1^
*T* = 296 K
0.17 × 0.15 × 0.15 mm

#### Data collection

Bruker APEXII CCD area-detector diffractometer
Absorption correction: multi-scan (*SADABS*; Bruker, 2001 ▶) *T*_min_ = 0.884, *T*_max_ = 0.897
7556 measured reflections
2744 independent reflections
2455 reflections with *I* > 2σ(*I*)
*R*_int_ = 0.026

#### Refinement

*R*[*F* ^2^ > 2σ(*F* ^2^)] = 0.029
*wR*(*F* ^2^) = 0.069
*S* = 1.03
2744 reflections
218 parameters
H-atom parameters constrained
Δρ_max_ = 0.29 e Å^−3^
Δρ_min_ = −0.31 e Å^−3^

### 

Data collection: *APEX2* (Bruker, 2007 ▶); cell refinement: *SAINT* (Bruker, 2007 ▶); data reduction: *SAINT*; program(s) used to solve structure: *SIR97* (Altomare *et al.*, 1999 ▶); program(s) used to refine structure: *SHELXTL* (Sheldrick, 2008 ▶); molecular graphics: *SHELXTL*; software used to prepare material for publication: *WinGX* (Farrugia, 1999 ▶).

## Supplementary Material

Crystal structure: contains datablock(s) global, I. DOI: 10.1107/S1600536811032168/nk2102sup1.cif

Structure factors: contains datablock(s) I. DOI: 10.1107/S1600536811032168/nk2102Isup2.hkl

Additional supplementary materials:  crystallographic information; 3D view; checkCIF report

## Figures and Tables

**Table 1:** Hydrogen-bond geometry (Å, °)

*D*—H⋯*A*	*D*—H	H⋯*A*	*D*⋯*A*	*D*—H⋯*A*
O1*W*—H1*W*⋯O2^i^	0.86	1.80	2.6388 (17)	164
N6—H6⋯O2^ii^	0.85	2.07	2.855 (2)	154
N8—H8⋯N7^iii^	0.86	2.20	3.018 (3)	160

Symmetry codes: (i) 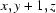; (ii) 
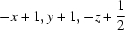
; (iii) 
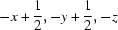
.

**Fig. 1.**
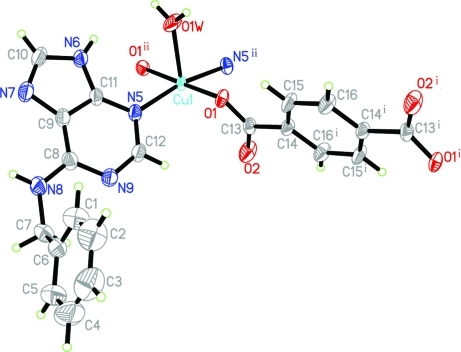
Anisotropic displacement ellipsoid plot of (I) at the 50% probability level. H atoms are represented by circles of arbitrary size. Symmetry code: (i)-x + 1, -y, -z + 1; (ii)-x + 1, y, -z + 1/2.

**Fig. 2.**
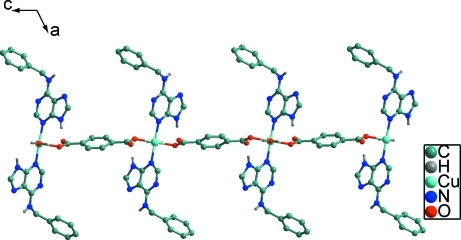
The one-dimensional chain structure of (I). Non-associative H atoms are omitted.

**Fig. 3.**
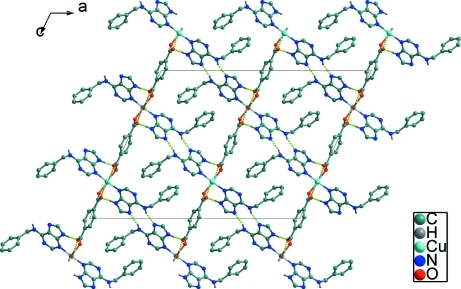
The packing diagram of (I) showing hydrogen bonding interactions (light blue dashed lines).

## supplementary crystallographic information

### Comment

Recently, biomolecules such as 2-amino-3-(4-aminophenyl)-propionic acid (Xie *et al.*, 2007), glycine and alanine(Lee *et al.*, 2008) and adenine (An *et al.*, 2009) were used to construct metal-organic frameworks (MOFs) due potential biomedical usefulness. During the synthesis of bio-MOFs using a biomolecule and Cu^II^ ion, the title compound (I) was obtained, and here its crystal structure is reported.

The asymmetric unit of (I) is composed of one Cu^II^ cation, one *N*^6^-benzyladenine molecule, half of benzene-1,4-dicarboxylate anion and one water molecule. As shown in Figure 1, the Cu^II^ ion is five-coordinated by two carboxylate O atoms from two different benzene-1,4-dicarboxylate ligands, two N atoms from two different *N*^6^-benzyladenine ligands and one water O atom in a square-pyramidal coordination environment. The Cu^II^ and water O atoms lie on a twofold rotation axis, and the benzene-1,4-dicarboxylate moiety lies on inversion center. The water O atom occupies the apical position and the basal plane is occupied by two O atoms and two N atoms. Each benzene-1,4- dicarboxylate anion acts as a bis-monodentate ligand that binds two Cu^II^ cations, forming an infinite chain extending parallel to [001] (Fig. 2). The *N*^6^-benzyladenine ligands are attached on both sides of the chain. The neighbouring chains are connected into two dimensional layers *via* O—H···O and N—H···O hydrogen bonds, and the adjacent layers are further packed *via* N—H···N hydrogen bonds into the three dimensional supramolecular architecture (Table 1, Fig. 3).

### Experimental

A mixture of benzene-1,4-dicarboxylate acid (0.017 g, 0.1 mmol), *N*^6^-benzyladenine (0.023 g, 0.1 mmol), and Cu(NO_3_)_2_.3H_2_O (0.024 g, 0.1 mmol) in H_2_O (10.0 ml) was placed in a 16 ml Teflon-lined stainless steel vessel and heated to 120 °C for 72 h, then cooled to room temperature at a rate of -5 °C/h. Afer filtration, dark blue block crystals are obtained.

### Refinement

All H atoms bonded to C and N atoms were added according to theoretical models, assigned isotropic displacement parameters and allowed to ride on their respective parent atoms [*U*_iso_(H) =1.2*U*_eq_(C)]. The H atoms attached to O atoms of the water were located from a difference Fourier map with the O—H distances being fixed at 0.85 Å and allowed to ride on their parent O atoms in the final cycles of refinement, with *U*_iso_(H) = 1.2*U*_eq_(O).

### Crystal data

**Table:**

[Cu(C_8_H_4_O_4_)(C_12_H_11_N_5_)_2_(H_2_O)]	*F*(000) = 1436
*M**_r_* = 696.18	*D*_x_ = 1.486 Mg m^−^^3^
Monoclinic, *C*2/*c*	Mo *K*α radiation, λ = 0.71073 Å
*a* = 28.171 (2) Å	Cell parameters from 3162 reflections
*b* = 5.554 (1) Å	θ = 3.0–27.3°
*c* = 22.102 (1) Å	µ = 0.76 mm^−^^1^
β = 115.868 (1)°	*T* = 296 K
*V* = 3111.6 (6) Å^3^	Block, blue
*Z* = 4	0.17 × 0.15 × 0.15 mm

### Data collection

**Table:**

Bruker APEXII CCD area-detector diffractometer	2744 independent reflections
Radiation source: fine-focus sealed tube	2455 reflections with *I* > 2σ(*I*)
graphite	*R*_int_ = 0.026
φ and ω scans	θ_max_ = 25.0°, θ_min_ = 1.6°
Absorption correction: multi-scan (*SADABS*; Bruker, 2001)	*h* = −30→33
*T*_min_ = 0.884, *T*_max_ = 0.897	*k* = −6→6
7556 measured reflections	*l* = −25→26

### Refinement

**Table:**

Refinement on *F*^2^	Primary atom site location: structure-invariant direct methods
Least-squares matrix: full	Secondary atom site location: difference Fourier map
*R*[*F*^2^ > 2σ(*F*^2^)] = 0.029	Hydrogen site location: inferred from neighbouring sites
*wR*(*F*^2^) = 0.069	H-atom parameters constrained
*S* = 1.03	*w* = 1/[σ^2^(*F*_o_^2^) + (0.0245*P*)^2^ + 4.4543*P*] where *P* = (*F*_o_^2^ + 2*F*_c_^2^)/3
2744 reflections	(Δ/σ)_max_ = 0.012
218 parameters	Δρ_max_ = 0.29 e Å^−^^3^
0 restraints	Δρ_min_ = −0.31 e Å^−^^3^

### Special details

**Table:**

Geometry. All e.s.d.'s (except the e.s.d. in the dihedral angle between two l.s. planes) are estimated using the full covariance matrix. The cell e.s.d.'s are taken into account individually in the estimation of e.s.d.'s in distances, angles and torsion angles; correlations between e.s.d.'s in cell parameters are only used when they are defined by crystal symmetry. An approximate (isotropic) treatment of cell e.s.d.'s is used for estimating e.s.d.'s involving l.s. planes.
Refinement. Refinement of *F*^2^ against ALL reflections. The weighted *R*-factor *wR* and goodness of fit *S* are based on *F*^2^, conventional *R*-factors *R* are based on *F*, with *F* set to zero for negative *F*^2^. The threshold expression of *F*^2^ > σ(*F*^2^) is used only for calculating *R*-factors(gt) *etc*. and is not relevant to the choice of reflections for refinement. *R*-factors based on *F*^2^ are statistically about twice as large as those based on *F*, and *R*- factors based on ALL data will be even larger.

### Fractional atomic coordinates and isotropic or equivalent isotropic displacement parameters (Å^2^)

**Table:**

	*x*	*y*	*z*	*U*_iso_*/*U*_eq_	
C1	0.21656 (11)	−0.0279 (5)	0.16879 (14)	0.0529 (7)	
H1	0.2405	0.0865	0.1682	0.063*	
C2	0.19178 (13)	0.0055 (6)	0.21029 (16)	0.0680 (9)	
H2	0.1992	0.1421	0.2372	0.082*	
C3	0.15662 (13)	−0.1605 (7)	0.21196 (17)	0.0706 (9)	
H3	0.1402	−0.1381	0.2400	0.085*	
C4	0.14592 (12)	−0.3596 (7)	0.17203 (17)	0.0688 (9)	
H4	0.1221	−0.4737	0.1731	0.083*	
C5	0.17008 (10)	−0.3938 (5)	0.12996 (14)	0.0533 (7)	
H5	0.1620	−0.5292	0.1025	0.064*	
C6	0.20617 (8)	−0.2284 (4)	0.12849 (11)	0.0375 (5)	
C7	0.23445 (9)	−0.2828 (4)	0.08544 (12)	0.0398 (6)	
H7A	0.2598	−0.4101	0.1070	0.048*	
H7B	0.2088	−0.3439	0.0424	0.048*	
C8	0.31312 (8)	−0.0374 (4)	0.10970 (10)	0.0305 (5)	
C9	0.34020 (8)	0.1452 (4)	0.09326 (10)	0.0302 (5)	
C10	0.36693 (9)	0.4238 (5)	0.05042 (11)	0.0424 (6)	
H10	0.3680	0.5461	0.0223	0.051*	
C11	0.39354 (8)	0.1695 (4)	0.13382 (9)	0.0264 (5)	
C12	0.39179 (8)	−0.1299 (4)	0.20000 (10)	0.0301 (5)	
H12	0.4091	−0.2269	0.2376	0.036*	
C13	0.49770 (7)	−0.0984 (4)	0.37039 (9)	0.0243 (4)	
C14	0.49924 (8)	−0.0458 (4)	0.43782 (9)	0.0249 (4)	
C15	0.48078 (9)	0.1719 (4)	0.44969 (10)	0.0321 (5)	
H15	0.4679	0.2879	0.4160	0.039*	
C16	0.48156 (9)	0.2161 (4)	0.51161 (10)	0.0331 (5)	
H16	0.4690	0.3622	0.5194	0.040*	
Cu1	0.5000	0.07414 (6)	0.2500	0.01886 (11)	
N5	0.42174 (6)	0.0364 (3)	0.18941 (8)	0.0254 (4)	
N6	0.41010 (7)	0.3488 (3)	0.10540 (8)	0.0344 (4)	
H6	0.4407	0.4096	0.1204	0.041*	
N7	0.32360 (7)	0.3089 (4)	0.04042 (9)	0.0402 (5)	
N8	0.26185 (7)	−0.0814 (4)	0.07311 (9)	0.0393 (5)	
H8	0.2441	0.0147	0.0405	0.047*	
N9	0.34090 (7)	−0.1755 (3)	0.16430 (9)	0.0326 (4)	
O1	0.48680 (5)	0.0769 (3)	0.32972 (6)	0.0249 (3)	
O2	0.50664 (7)	−0.3073 (3)	0.35810 (7)	0.0418 (4)	
O1W	0.5000	0.4643 (4)	0.2500	0.0417 (6)	
H1W	0.4975	0.5551	0.2799	0.050*	

### Atomic displacement parameters (Å^2^)

**Table:**

	*U*^11^	*U*^22^	*U*^33^	*U*^12^	*U*^13^	*U*^23^
C1	0.0511 (16)	0.0493 (17)	0.0595 (17)	−0.0067 (13)	0.0253 (14)	−0.0023 (14)
C2	0.079 (2)	0.067 (2)	0.0635 (19)	0.0095 (18)	0.0359 (17)	−0.0076 (16)
C3	0.071 (2)	0.089 (3)	0.069 (2)	0.0161 (19)	0.0456 (18)	0.0113 (19)
C4	0.0547 (18)	0.085 (2)	0.080 (2)	−0.0095 (17)	0.0410 (17)	0.0169 (19)
C5	0.0473 (15)	0.0555 (18)	0.0581 (17)	−0.0127 (13)	0.0238 (13)	0.0005 (14)
C6	0.0275 (11)	0.0427 (14)	0.0368 (12)	−0.0031 (10)	0.0090 (10)	0.0071 (11)
C7	0.0284 (11)	0.0450 (15)	0.0417 (13)	−0.0087 (11)	0.0113 (10)	−0.0022 (11)
C8	0.0247 (10)	0.0400 (13)	0.0259 (11)	−0.0013 (10)	0.0102 (9)	0.0007 (10)
C9	0.0254 (11)	0.0389 (13)	0.0231 (10)	−0.0004 (9)	0.0077 (9)	0.0044 (9)
C10	0.0345 (12)	0.0519 (15)	0.0331 (12)	−0.0023 (12)	0.0077 (10)	0.0198 (12)
C11	0.0234 (10)	0.0356 (12)	0.0189 (10)	−0.0008 (9)	0.0081 (8)	0.0010 (9)
C12	0.0277 (11)	0.0390 (13)	0.0227 (10)	0.0022 (9)	0.0100 (9)	0.0069 (9)
C13	0.0257 (10)	0.0324 (12)	0.0162 (9)	0.0004 (9)	0.0105 (8)	−0.0013 (9)
C14	0.0355 (11)	0.0261 (11)	0.0168 (9)	0.0004 (9)	0.0148 (8)	−0.0002 (8)
C15	0.0530 (14)	0.0271 (11)	0.0196 (10)	0.0082 (10)	0.0190 (10)	0.0065 (9)
C16	0.0564 (14)	0.0246 (11)	0.0254 (11)	0.0086 (10)	0.0244 (10)	0.0015 (9)
Cu1	0.02049 (18)	0.02546 (19)	0.01114 (16)	0.000	0.00736 (13)	0.000
N5	0.0219 (8)	0.0360 (10)	0.0180 (8)	−0.0006 (8)	0.0085 (7)	0.0026 (7)
N6	0.0238 (9)	0.0454 (12)	0.0270 (9)	−0.0074 (8)	0.0047 (8)	0.0096 (8)
N7	0.0292 (10)	0.0512 (13)	0.0321 (10)	−0.0013 (9)	0.0058 (8)	0.0160 (9)
N8	0.0237 (9)	0.0524 (13)	0.0346 (10)	−0.0063 (9)	0.0060 (8)	0.0112 (10)
N9	0.0255 (9)	0.0416 (11)	0.0288 (10)	−0.0026 (8)	0.0100 (8)	0.0075 (8)
O1	0.0310 (7)	0.0314 (8)	0.0161 (6)	0.0058 (6)	0.0138 (6)	0.0044 (6)
O2	0.0755 (12)	0.0309 (9)	0.0264 (8)	0.0113 (8)	0.0291 (8)	−0.0011 (7)
O1W	0.0846 (18)	0.0243 (12)	0.0234 (11)	0.000	0.0305 (12)	0.000

### Geometric parameters (Å, °)

**Table:**

C1—C6	1.375 (4)	C11—N5	1.355 (2)
C1—C2	1.386 (4)	C11—N6	1.364 (3)
C1—H1	0.9300	C12—N9	1.325 (3)
C2—C3	1.366 (4)	C12—N5	1.339 (3)
C2—H2	0.9300	C12—H12	0.9300
C3—C4	1.364 (5)	C13—O2	1.242 (2)
C3—H3	0.9300	C13—O1	1.269 (2)
C4—C5	1.384 (4)	C13—C14	1.501 (2)
C4—H4	0.9300	C14—C16^i^	1.382 (3)
C5—C6	1.381 (3)	C14—C15	1.386 (3)
C5—H5	0.9300	C15—C16	1.381 (3)
C6—C7	1.514 (3)	C15—H15	0.9300
C7—N8	1.451 (3)	C16—C14^i^	1.382 (3)
C7—H7A	0.9700	C16—H16	0.9300
C7—H7B	0.9700	Cu1—O1^ii^	1.9531 (12)
C8—N8	1.334 (3)	Cu1—O1	1.9531 (12)
C8—N9	1.354 (3)	Cu1—N5^ii^	2.0301 (16)
C8—C9	1.409 (3)	Cu1—N5	2.0301 (15)
C9—C11	1.380 (3)	Cu1—O1W	2.167 (2)
C9—N7	1.390 (3)	N6—H6	0.8474
C10—N7	1.308 (3)	N8—H8	0.8600
C10—N6	1.356 (3)	O1W—H1W	0.8593
C10—H10	0.9300		
			
C6—C1—C2	120.7 (3)	N9—C12—H12	115.5
C6—C1—H1	119.6	N5—C12—H12	115.5
C2—C1—H1	119.6	O2—C13—O1	124.85 (17)
C3—C2—C1	120.5 (3)	O2—C13—C14	118.56 (18)
C3—C2—H2	119.7	O1—C13—C14	116.59 (18)
C1—C2—H2	119.7	C16^i^—C14—C15	119.42 (17)
C2—C3—C4	119.2 (3)	C16^i^—C14—C13	120.10 (18)
C2—C3—H3	120.4	C15—C14—C13	120.47 (18)
C4—C3—H3	120.4	C16—C15—C14	119.85 (19)
C3—C4—C5	120.8 (3)	C16—C15—H15	120.1
C3—C4—H4	119.6	C14—C15—H15	120.1
C5—C4—H4	119.6	C15—C16—C14^i^	120.73 (19)
C6—C5—C4	120.5 (3)	C15—C16—H16	119.6
C6—C5—H5	119.8	C14^i^—C16—H16	119.6
C4—C5—H5	119.8	O1^ii^—Cu1—O1	179.10 (9)
C1—C6—C5	118.3 (2)	O1^ii^—Cu1—N5^ii^	90.94 (6)
C1—C6—C7	123.2 (2)	O1—Cu1—N5^ii^	89.15 (6)
C5—C6—C7	118.5 (2)	O1^ii^—Cu1—N5	89.15 (6)
N8—C7—C6	115.7 (2)	O1—Cu1—N5	90.94 (6)
N8—C7—H7A	108.4	N5^ii^—Cu1—N5	168.16 (10)
C6—C7—H7A	108.4	O1^ii^—Cu1—O1W	89.55 (4)
N8—C7—H7B	108.4	O1—Cu1—O1W	89.55 (4)
C6—C7—H7B	108.4	N5^ii^—Cu1—O1W	95.92 (5)
H7A—C7—H7B	107.4	N5—Cu1—O1W	95.92 (5)
N8—C8—N9	119.24 (19)	C12—N5—C11	111.72 (16)
N8—C8—C9	122.70 (19)	C12—N5—Cu1	122.80 (13)
N9—C8—C9	118.05 (18)	C11—N5—Cu1	125.48 (13)
C11—C9—N7	110.70 (18)	C10—N6—C11	106.49 (17)
C11—C9—C8	117.49 (19)	C10—N6—H6	126.1
N7—C9—C8	131.77 (19)	C11—N6—H6	127.2
N7—C10—N6	114.0 (2)	C10—N7—C9	103.31 (17)
N7—C10—H10	123.0	C8—N8—C7	123.45 (19)
N6—C10—H10	123.0	C8—N8—H8	118.3
N5—C11—N6	129.28 (18)	C7—N8—H8	118.3
N5—C11—C9	125.26 (19)	C12—N9—C8	118.51 (18)
N6—C11—C9	105.46 (17)	C13—O1—Cu1	123.41 (12)
N9—C12—N5	128.93 (19)	Cu1—O1W—H1W	126.0
			
C6—C1—C2—C3	0.1 (5)	N6—C11—N5—Cu1	1.5 (3)
C1—C2—C3—C4	−0.3 (5)	C9—C11—N5—Cu1	−179.13 (16)
C2—C3—C4—C5	−0.3 (5)	O1^ii^—Cu1—N5—C12	131.20 (16)
C3—C4—C5—C6	1.1 (5)	O1—Cu1—N5—C12	−49.70 (16)
C2—C1—C6—C5	0.7 (4)	N5^ii^—Cu1—N5—C12	40.65 (16)
C2—C1—C6—C7	−176.0 (3)	O1W—Cu1—N5—C12	−139.35 (16)
C4—C5—C6—C1	−1.2 (4)	O1^ii^—Cu1—N5—C11	−47.81 (16)
C4—C5—C6—C7	175.6 (2)	O1—Cu1—N5—C11	131.29 (16)
C1—C6—C7—N8	−17.5 (3)	N5^ii^—Cu1—N5—C11	−138.35 (16)
C5—C6—C7—N8	165.8 (2)	O1W—Cu1—N5—C11	41.65 (16)
N8—C8—C9—C11	−178.2 (2)	N7—C10—N6—C11	0.0 (3)
N9—C8—C9—C11	0.5 (3)	N5—C11—N6—C10	179.7 (2)
N8—C8—C9—N7	−0.9 (4)	C9—C11—N6—C10	0.3 (2)
N9—C8—C9—N7	177.8 (2)	N6—C10—N7—C9	−0.2 (3)
N7—C9—C11—N5	−179.9 (2)	C11—C9—N7—C10	0.4 (3)
C8—C9—C11—N5	−2.1 (3)	C8—C9—N7—C10	−177.0 (2)
N7—C9—C11—N6	−0.4 (2)	N9—C8—N8—C7	−4.9 (3)
C8—C9—C11—N6	177.40 (19)	C9—C8—N8—C7	173.9 (2)
O2—C13—C14—C16^i^	10.3 (3)	C6—C7—N8—C8	95.4 (3)
O1—C13—C14—C16^i^	−170.24 (19)	N5—C12—N9—C8	−1.4 (3)
O2—C13—C14—C15	−168.8 (2)	N8—C8—N9—C12	179.8 (2)
O1—C13—C14—C15	10.7 (3)	C9—C8—N9—C12	1.0 (3)
C16^i^—C14—C15—C16	−0.2 (4)	O2—C13—O1—Cu1	−16.8 (3)
C13—C14—C15—C16	178.84 (19)	C14—C13—O1—Cu1	163.75 (12)
C14—C15—C16—C14^i^	0.2 (4)	O1^ii^—Cu1—O1—C13	−157.61 (15)
N9—C12—N5—C11	0.0 (3)	N5^ii^—Cu1—O1—C13	−61.68 (15)
N9—C12—N5—Cu1	−179.12 (17)	N5—Cu1—O1—C13	106.48 (15)
N6—C11—N5—C12	−177.6 (2)	O1W—Cu1—O1—C13	−157.61 (14)
C9—C11—N5—C12	1.8 (3)		

Symmetry codes: (i) −*x*+1, −*y*, −*z*+1; (ii) −*x*+1, *y*, −*z*+1/2.

### Hydrogen-bond geometry (Å, °)

**Table:**

*D*—H···*A*	*D*—H	H···*A*	*D*···*A*	*D*—H···*A*
O1W—H1W···O2^iii^	0.86	1.80	2.6388 (17)	164.
N6—H6···O2^iv^	0.85	2.07	2.855 (2)	154.
N8—H8···N7^v^	0.86	2.20	3.018 (3)	160.

Symmetry codes: (iii) *x*, *y*+1, *z*; (iv) −*x*+1, *y*+1, −*z*+1/2; (v) −*x*+1/2, −*y*+1/2, −*z*.
